# Idiopathic CD4 lymphocytopenia presenting as refractory cryptococcal meningitis

**DOI:** 10.4103/0972-2327.64646

**Published:** 2010

**Authors:** A. Sharma, V. Lal, M. Modi, D. Khurana, S. Bal, S. Prabhakar

**Affiliations:** Department of Neurology, Postgraduate Institute of Medical Education and Research, Chandigarh-160 012, India

**Keywords:** Cryptococcus, CD4, idiopathic

## Abstract

Idiopathic CD4 T-lymphocytopenia (ICL) is a syndrome characterized by depletion of CD4 T-cells without evidence of human immunodeficiency virus (HIV) infection. There are a few reported cases of ICL associated with different diseases and clinical conditions, most commonly the opportunistic infections like Tuberculosis, fungal and parasitic diseases which are also seen in HIV-positive patients. We report a case without risk factors or laboratory evidence of HIV infection who presented with refractory cryptococcal meningitis and was found to have ICL.

## Introduction

Idiopathic CD4 lymphocytopenia (ICL) was defined by the United States Centers for Disease Control and Prevention (CDC) as a clinical condition in patients with depressed numbers of circulating CD4 T lymphocytes (<300 cells/μl or <20% of total T cells) at a minimum of two separate time points at least 6 weeks apart, with no laboratory evidence of infection with human HIV-1 or HIV-2, and the absence of any defined immunodeficiency or therapy associated with depressed levels of CD4 T cells.[1] The provisional case definition by the CDC therefore also permits the inclusion of patients with panlymphocytopenia and normal CD4:CD8 ratio, although most of the published cases had a severely inverted CD4:CD8 ratio. There are case reports of ICL associated with different diseases and clinical conditions, mostly fungal, parasitic, and viral infections.[2]

## Case Report

**Figure 1:**
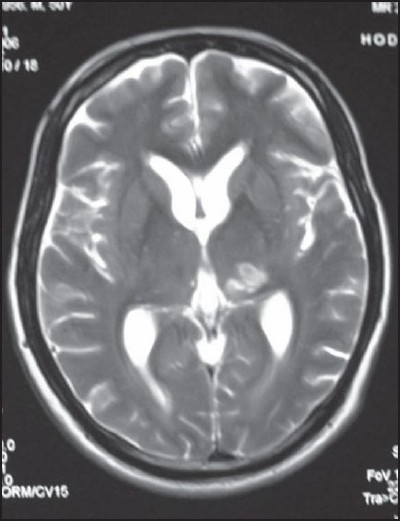
MRI brain, axial view, showing T2 hyperintensity in the left thalamus that is extending into the corona radiata

A 50-year-old male with no history of any significant illness in the past presented with complaints of fever, holocranial headache of 2 months' duration, one episode of a left-sided focal seizure with secondary generalization, and decreased sensation to touch in the right half of body for 15 days. He had no risk factors for HIV infection and had not received any immunosuppressive therapy. There was no history of weakness in any limb, no cranial nerve deficits, and no evidence of cognitive decline. On examination, he was averagely built and well nourished, with normal pulse and blood pressure and no clinical lymphadenopathy. Neurological examination revealed bilateral papilledema; exaggerated deep tendon reflexes in the right upper and lower limbs; flexor plantars; and decreased pain and temperature sensation in the right half of the body, with sparing of the face. The neurological examination was otherwise normal. Plain magnetic resonance imaging (MRI) brain revealed a T2 hyperintensity in the left thalamus, extending to involve the left corona radiata [Figure 1]. There was no meningeal enhancement on contrast-enhanced MRI. cerebrospinal fluid (CSF) study revealed no cells, sugar of 17 mg/dl (corresponding blood sugar: 80 mg/dl), proteins 125 mg, positive cryptococcal antigen (with titer of 1:8192), and positive fungal culture for *Cryptococcus neoformans*. CSF studies were negative for toxoplasma antigens. Further workup to rule out an underlying immunocompromised state was negative for HIV (1 and 2). The patient was started on intravenous amphotericin B, 0.5–1.0 mg/kg (cumulative dose of 4 gm). After 8 weeks of therapy cryptola titer showed decreased values (1:1024) and fungal culture was negative for cryptococcus. The patient improved clinically and showed resolving papilledema on fundus examination. He was discharged on oral fluconazole 400 mg/day.

Two weeks later he was readmitted in the Emergency with complaints of severe holocranial headache and vomiting. Fundus examination revealed papilledema. No other localizing signs were present on neurological examination. Computed tomographic (CT) scan of the brain during this admission showed hydrocephalus; a repeat CSF study showed low sugar (26 mg/dl), raised protein (100 mg%), and raised cryptococcal antigen titer (1:2048); CSF culture showed growth of *C neoformans* resistant to fluconazole. CSF study for malignant cytology and repeat serology for HIV was negative. CT scan (chest and abdomen) was normal. The patient was restarted on intravenous amphotericin B. CD4 counts were asked for to rule out an underlying immunocompromised state and was found to be low (203/μl), with an inverted CD4:CD8 ratio of 0.74 (normal: 1.4–1.6). After 6 weeks of treatment, the patient showed improvement clinically and there was a gradual decline in serial cryptococcal antigen titers (1:128). CD4 counts, however, further declined to 99/μl The patient was continued on parenteral amphotericin B, with oral voriconazole 100 mg/day being added on the basis of the culture and sensitivity report. After 8 weeks, amphotericin B (cumulative dose of 4.5 gm) was stopped and the patient was discharged on oral voriconazole 100 mg/day. He is on regular follow-up. At present he is asymptomatic on oral voriconazole, with a CSF cryptococcal antigen titer of 1: <32 and a low CD4 count of 124/ μl.

## Discussion

Unlike transient CD4 lymphocytopenia, which is common and has been estimated to occur in 0.4–4.1% healthy HIV-negative individuals at any given time, ICL in an immunocompetent adult is very rare.[3] In the largest survey to date, involving 47 ICL patients, it was found to be more common in males (M:F ratio of 1.6:1); it most often occurred in the age-group of 43 ± 14 years (range 17–78 years).[4] A few familial cases have been reported. No evidence of sexual transmission has been found.[5]

The most important differential diagnosis of CD4 lymphocytopenia is HIV infection. Common pathogenic and opportunistic bacterial, viral (hepatitis B, Ebstein-Barr virus, and cytomegalovirus), parasitic, and fungal diseases may depress CD4 cell counts, but usually without inversion of the CD4:CD8 ratio.[6] The changes associated with these infections are mostly transient and therefore probably 'physiologic' responses to an alteration of the cytokine and inflammatory environment.[7] Several hematological malignancies like non-Hodgkin lymphoma [large cell lymphoma, mucosa-associated lymphatic tissue (MALT) lymphoma, and Burkitt lymphoma],[8] mycosis fungoides, and the myelodysplastic syndrome (refractory anemia)[9] may cause CD4 lymphocytopenia with a normal CD4:CD8 ratio. Autoimmune diseases like primary Sjögren syndrome[10] and systemic lupus erythematosus (SLE)[11] can also cause CD4 lymphocytopenia. Chemotherapeutic drugs like cyclophosphamide, and less often methotrexate and azathioprine, have also been found to be associated with CD4 lymphocytopenia with normal CD4:CD8 ratios.[12] Cryptococcal meningitis is the most frequent and devastating fungal infection of the central nervous system in immunocompromised patients, mainly those with impaired cell-mediated immunity. Meningitis can relapse or can be resistant even after successful therapy, as in this patient, and resistance seems to be caused by the persistence of the original infecting strain.[13,14] Besides *C neoformans,* other bacterial *(Fusobacterium nucleatum, Mycobacterium tuberculosis, Mycobacterium avium intracellulare),* viral (cytomegalovirus, herpes simplex virus, human papilloma virus); fungal (aspergillus sp, *Candida albicans, Histoplasma capsulatum);* and parasitic (toxoplasma) infections have been found to be associated with ICL with an inverted CD4:CD8 ratio.[15] The few investigations in the pathogenesis of ICL suggest a diminished generation of T cell precursors and a decreased clonogenic capacity of bone marrow progenitors, which may be due to a disturbed cytokine environment with, for example, increased tumor necrosis factor-α (TNF-α) and decreased IL-2 levels.[16] CD4 T cell lymphocytopenia can be the result of cryptococcal infection alone, but our patient had a sustained CD4 T cell lymphopenia even after his cryptococcal meningitis was cured. This finding argues against possible secondary CD4 T cell lymphopenia. The cryptococcal meningitis in this case was an opportunistic infection, which was secondary to the CD4 depletion. Because of sustained CD4 T lymphocyte depletion, the lack of serological evidence of HIV infection, and the absence of any immunodeficiency or immunosuppresive therapy associated with T cell depletion, our patient met the existing criteria for ICL. The treatment of CD4 lymphocytopenia includes therapy of underlying conditions; treatment and prophylaxis of secondary complications, especially of opportunistic infections; and some experimental approaches like interleukin 2[17] and interleukin 7[18] therapy to enhance CD4 T cell counts. Allogeneic bone marrow transplantation (BMT) was performed in one ICL patient for a concomitant aplastic anemia, and this resulted in an improvement in the CD4 T cell counts[19]

## Conclusion

Idiopathic CD4 lymphocytopenia should be considered in all immunocompetent patients presenting with cryptococcal meningitis. It is thought to be reasonable to follow the treatment recommendations for HIV-infected patients with cryptococcal meningitis and to continue fluconazole or voriconazole for life in patients with CD4 lymphocytopenia who are negative for HIV infection. This condition, although rare, merits clinical suspicion in non – immunodeficient patients presenting with opportunistic infections.
